# Supplementary material for: Gene-signature-derived IC50s/EC50s reflect the potency of causative upstream targets and downstream phenotypes
Source: Sci Rep. 2020 Jun 15;10:9670. doi: 10.1038/s41598-020-66533-5 (PMC7295968; doi:10.1038/s41598-020-66533-5)
Supplement: Supplementary file 1 — Supplementary Information. [file 41598_2020_66533_MOESM1_ESM.pdf]

Supplementary Information for

Gene-signatures predict biologically relevant dose-response potencies in phenotypic assays

Steffen Renner[1*], Christian Bergsdorf[1], Rochdi Bouhelal[1], Magdalena Koziczak-Holbro[2], Andrea Marco Amati[1,6], Valerie Techer-Etienne[1], Ludivine Flotte[2], Nicole Reymann[1], Karen Kapur[3], Sebastian Hoersch[3], Edward J. Oakeley[4], Ansgar Schuffenhauer[1], Hanspeter Gubler[3], Eugen Lounkine[5,7], Pierre Farmer[1*]

1 Chemical Biology & Therapeutics, Novartis Institutes for Biomedical Research, Basel 4056, Switzerland

2 Musculoskeletal, NIBR, Basel, Switzerland

3 NIBR Informatics, NIBR, Basel, Switzerland

4 ASI, NIBR, Basel, Switzerland

5 Chemical Biology & Therapeutics, NIBR, 181 Massachusetts Avenue, Cambridge, MA 02139, USA

6 current address: Department of Chemistry & Biochemistry, University of Bern, Freiestrasse 3, 3012 Bern, Switzerland

7 current address: Modeling and Informatics, Merck & Co., Inc., 33 Avenue Louis Pasteur, Boston, MA 02115, USA

* Corresponding authors:

Steffen Renner: steffen.renner@novartis.com

Pierre Farmer: pierre.farmer@novartis.com

Supplementary Information

Supplementary Table 1: Genes selected for quantification of beta agonist potencies.

**Gene symbol**	**Gene id**	**Name**	**Set**	**Comment**
CCL22	6367	C-C motif chemokine ligand 22	1	
CD55	1604	CD55 molecule (Cromer blood group)	1	
DKK2	27123	dickkopf WNT signaling pathway inhibitor 2	1	
DOCK4	9732	dedicator of cytokinesis 4	1	
DUSP4	1846	dual specificity phosphatase 4	1	
IRF8	3394	interferon regulatory factor 8	1	
NR4A1	3164	nuclear receptor subfamily 4 group A member 1	1	
TBP	6908	TATA-box binding protein	1	Housekeeper
NR4A3	8013	nuclear receptor subfamily 4 group A member 3	2	
PDE4B	5142	phosphodiesterase 4B	2	
PPARGC1B	133522	PPARG coactivator 1 beta	2	
SGK1	6446	serum/glucocorticoid regulated kinase 1	2	
THBS1	6908	TATA-box binding protein	2	
TOB1	7057	thrombospondin 1	2	
VEGFA	10140	transducer of ERBB2, 1	2	
TBP	7422	vascular endothelial growth factor A	2	Housekeeper

Supplementary Table 2: Overview over compounds used in THP1 experiments, comparing cAMP and QuantiGene readouts.

Compound	MOA	Inchi_key	Smiles	EC_{50} repl1	Amax repl1	EC_{50} repl2	Amax repl2
(+/-)-isoproterenol	beta agonist	JWZZKOKVBUJMES-UHFFFAOYSA-N	CC(C)NCC(O)c1ccc(O)c(O)c1	0.00008	109	< 0.00001	110
terbutaline	beta agonist	XWTYSIMOBUGWOL-UHFFFAOYSA-N	CC(C)(C)NCC(O)c1cc(O)cc(O)c1	0.713	101	0.202	98
fenoterol	beta agonist	LSLYOANBFKQKPT-UHFFFAOYSA-N	CC(Cc1ccc(O)cc1)NCC(O)c2cc(O)cc(O)c2	0.0036	106	0.0013	111
adrenaline	beta agonist	UCTWMZQNUQWSLP-VIFPVBQESA-N	CNC[C@H](O)c1ccc(O)c(O)c1	0.139	106	0.0171	115
metaproterenol	beta agonist	LMOINURANNBYCM-UHFFFAOYSA-N	CC(C)NCC(O)c1cc(O)cc(O)c1	0.0036	108	0.0141	111
isoetharine	beta agonist	HUYWAWARQUIQLE-UHFFFAOYSA-N	CCC(NC(C)C)C(O)c1ccc(O)c(O)c1	0.0012	93	0.0115	108
BRL 37344	beta agonist	ZGGNJJJYUVRADP-UHFFFAOYSA-N	CC(Cc1ccc(cc1)OCC(O)=O)NCC(O)c2cccc(Cl)c2	0.0282	84	0.0984	91
ritodrine	beta agonist	IOVGROKTTNBUGK-SJCJKPOMSA-N	C[C@H](NCCc1ccc(O)cc1)[C@H](O)c2ccc(O)cc2	0.846	91	0.51	83
norepinephrine	beta agonist	SFLSHLFXELFNJZ-QMMMGPOBSA-N	NC[C@H](O)c1ccc(O)c(O)c1	0.0002792	109	0.0005829	115
procaterol	beta agonist	FKNXQNWAXFXVNW-BLLLJJGKSA-N	CC[C@H](NC(C)C)[C@H](O)c1ccc(O)c2NC(=O)C=Cc12	0.008542	93	0.00013	102
dobutamine	beta agonist	JRWZLRBJNMZMFE-UHFFFAOYSA-N	CC(CCc1ccc(O)cc1)NCCc2ccc(O)c(O)c2	0.0008564	106	0.00242	106
abediterol	beta agonist	SFYAXIFVXBKRPK-QFIPXVFZSA-N	O[C@@H](CNCCCCCCOCC(F)(F)c1ccccc1)c2ccc(O)c3NC(=O)C=Cc23	< 0.00001	101	< 0.00001	105
olodaterol	beta agonist	COUYJEVMBVSIHV-SFHVURJKSA-N	COc3ccc(CC(C)(C)NC[C@H](O)c2cc(O)cc1NC(=O)COc12)cc3	0.00001869	97	< 0.00001	102
batefenterol	beta agonist	URWYQGVSPQJGGB-DHUJRADRSA-N	COc4cc(NC(=O)CCN1CCC(CC1)OC(=O)Nc2ccccc2c3ccccc3)c(Cl)cc4CNC[C@H](O)c5ccc(O)c6NC(=O)C=Cc56	0.0021	101	1.89E-04	101
vilanterol	beta agonist	DAFYYTQWSAWIGS-DEOSSOPVSA-N	OCc1cc(ccc1O)[C@@H](O)CNCCCCCCOCCOCc2c(Cl)cccc2Cl	0.00001721	96	< 0.00001	98
tulobuterol	beta agonist	YREYLAVBNPACJM-UHFFFAOYSA-N	CC(C)(C)NCC(O)c1ccccc1Cl	0.016	68	0.0366	50
salbutamol	beta agonist	NDAUXUAQIAJITI-UHFFFAOYSA-N	CC(C)(C)NCC(O)c1ccc(O)c(CO)c1	0.02	89	0.004285	88
zinterol	beta agonist	XJBCFFLVLOPYBV-UHFFFAOYSA-N	CC(C)(Cc1ccccc1)NCC(O)c2ccc(O)c(c2)NS(C)(=O)=O	< 0.00001	99	0.00004344	95
tretoquinol	beta agonist	RGVPOXRFEPSFGH-UHFFFAOYSA-N	COc3cc(CC1NCCc2cc(O)c(O)cc12)cc(OC)c3OC	0.00005932	101	< 0.00001	105
dopexamine	beta agonist	RYBJORHCUPVNMB-UHFFFAOYSA-N	Oc2ccc(CCNCCCCCCNCCc1ccccc1)cc2O	4.1	56	5.837	62
formoterol	beta agonist	BPZSYCZIITTYBL-YJYMSZOUSA-N	COc2ccc(C[C@@H](C)NC[C@H](O)c1ccc(O)c(c1)NC=O)cc2	0.00001267	106	0.0000231	109
forskolin	AC activator	OHCQJHSOBUTRHG-KENMSXPPSA-N	CC(=O)OC2C(O)[C@H]1C(C)(C)CC[C@H](O)[C@]1(C)[C@@]3(O)C(=O)C[C@](C)(C=C)O[C@]23C	6.283	90	3.757	104
N-alpha-methylhistamine	histamine H3 antagonist	PHSPJQZRQAJPPF-UHFFFAOYSA-N	CNCCC1=CNC=N1	0.161	98	0.199	102
CGP-20712A	beta-1 antagonist	GKJZEKSHCJELPL-UHFFFAOYSA-N	CN1C=C(N=C1c2ccc(cc2)OCC(O)CNCCOc3ccc(O)c(c3)C(N)=O)C(F)(F)F	> 100	-17	> 100	-19

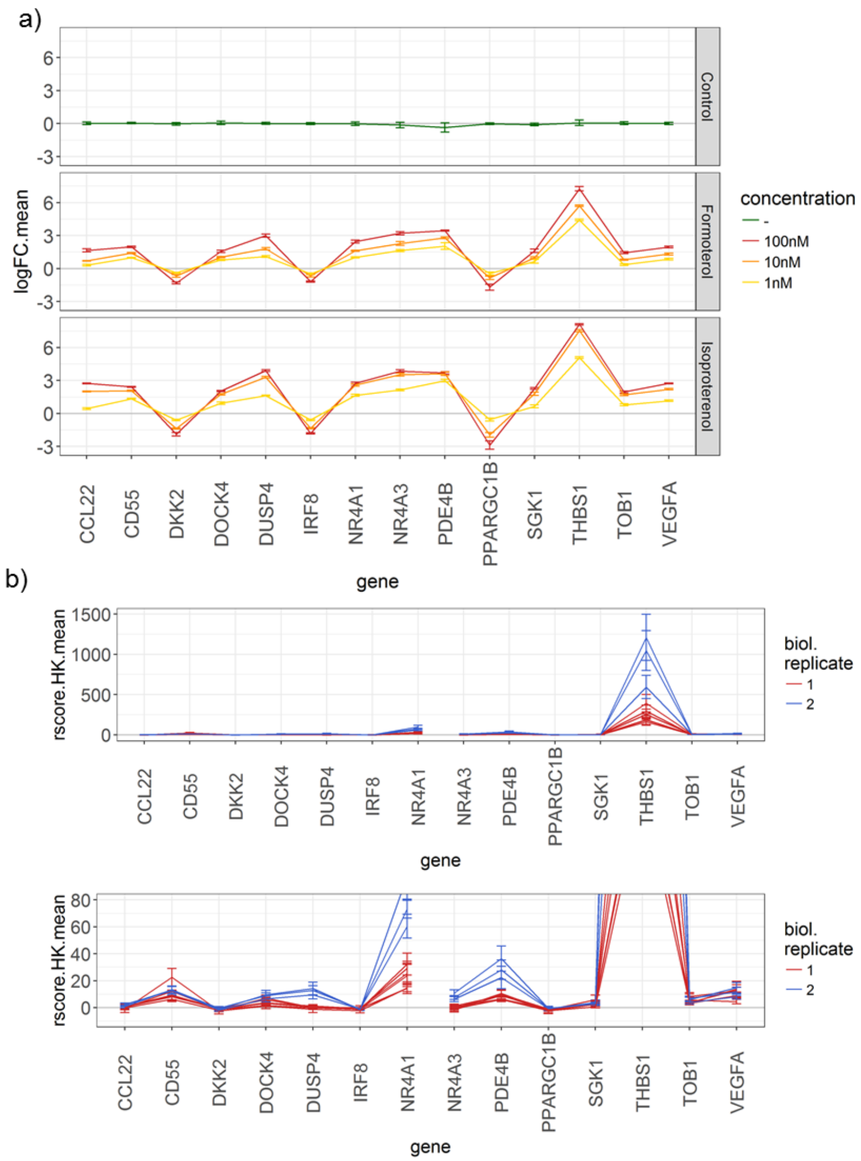

Supplementary Figure 1: Validation of the beta agonists gene-signature. a) qPCR results for DMSO, formoterol and isoproterenol demonstrate dose dependent effect on genes after 4h incubation. b) QuantiGene Plex results for the two gene-signatures for 10uM of isoproterenol after 4h incubation, shown at two different scales: upper = full scale with THBS1 having a much stronger response than the other genes, and lower = y-axis cut at 80 to visualize the genes with lower variance. Shown are mean standard deviations of the rscore_HK values of active control wells of each plate.

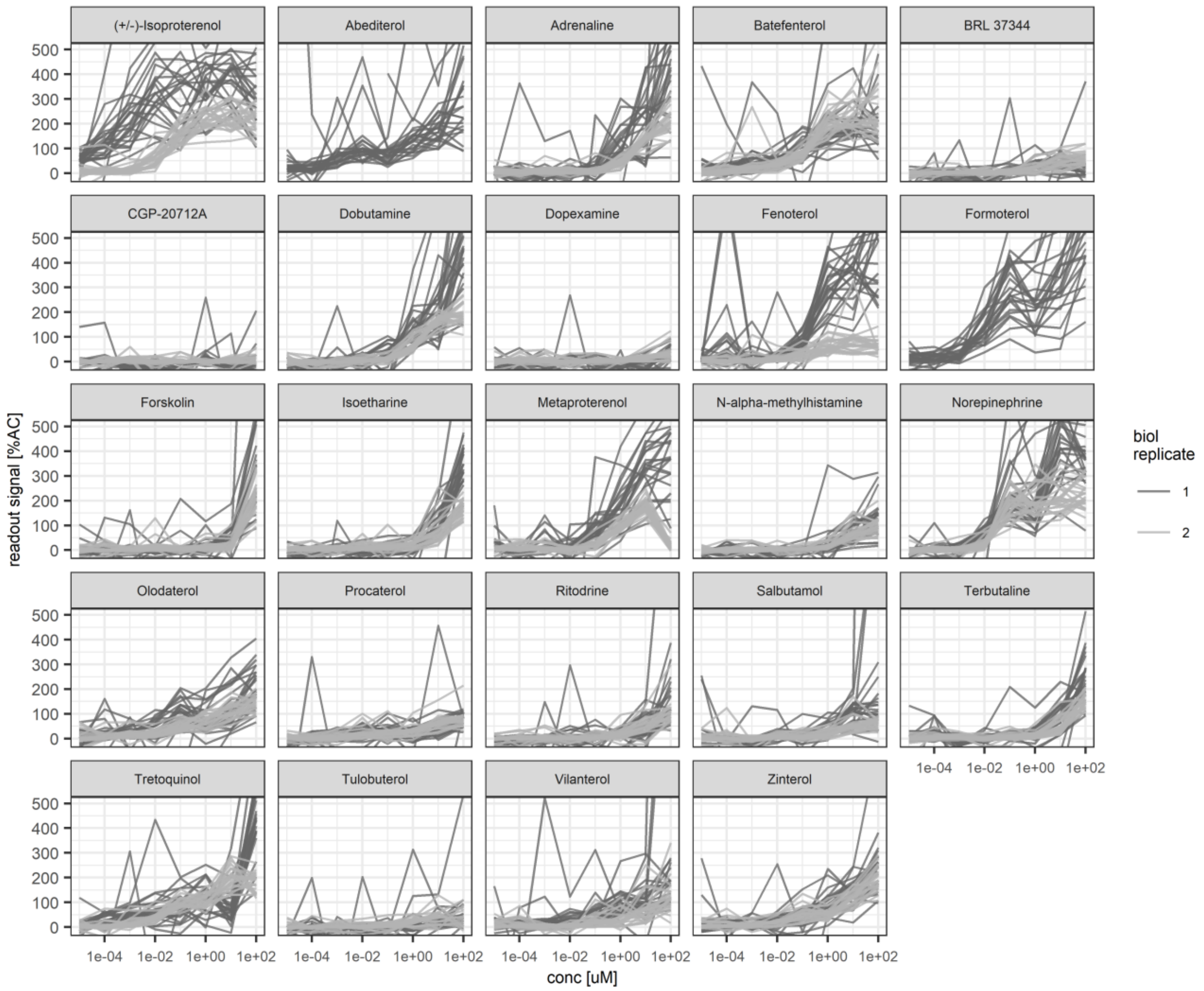

Supplementary Figure 2: Dose-response of genes for each compound in the beta agonists dataset.

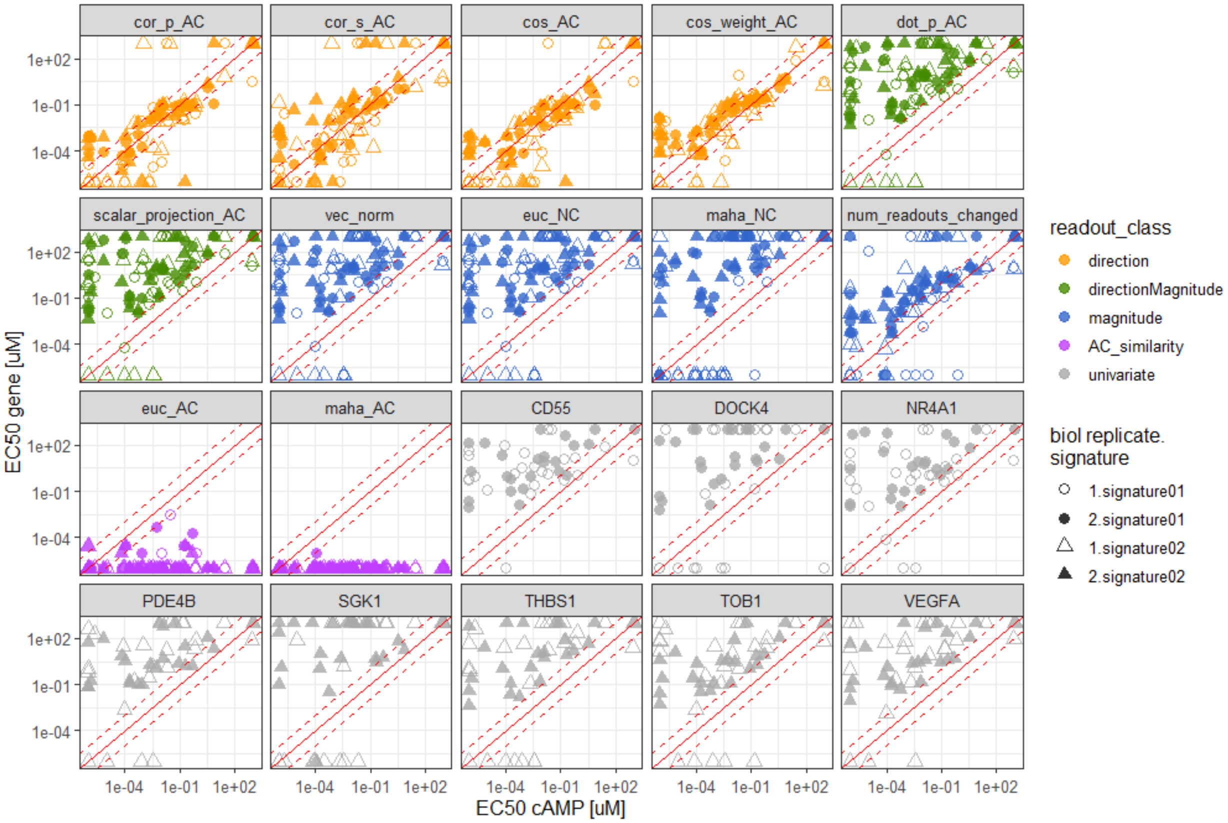

Supplementary Figure 3: Correlation of gene and gene-signature EC_{50}s with cAMP EC_{50}s of the beta agonist dataset.

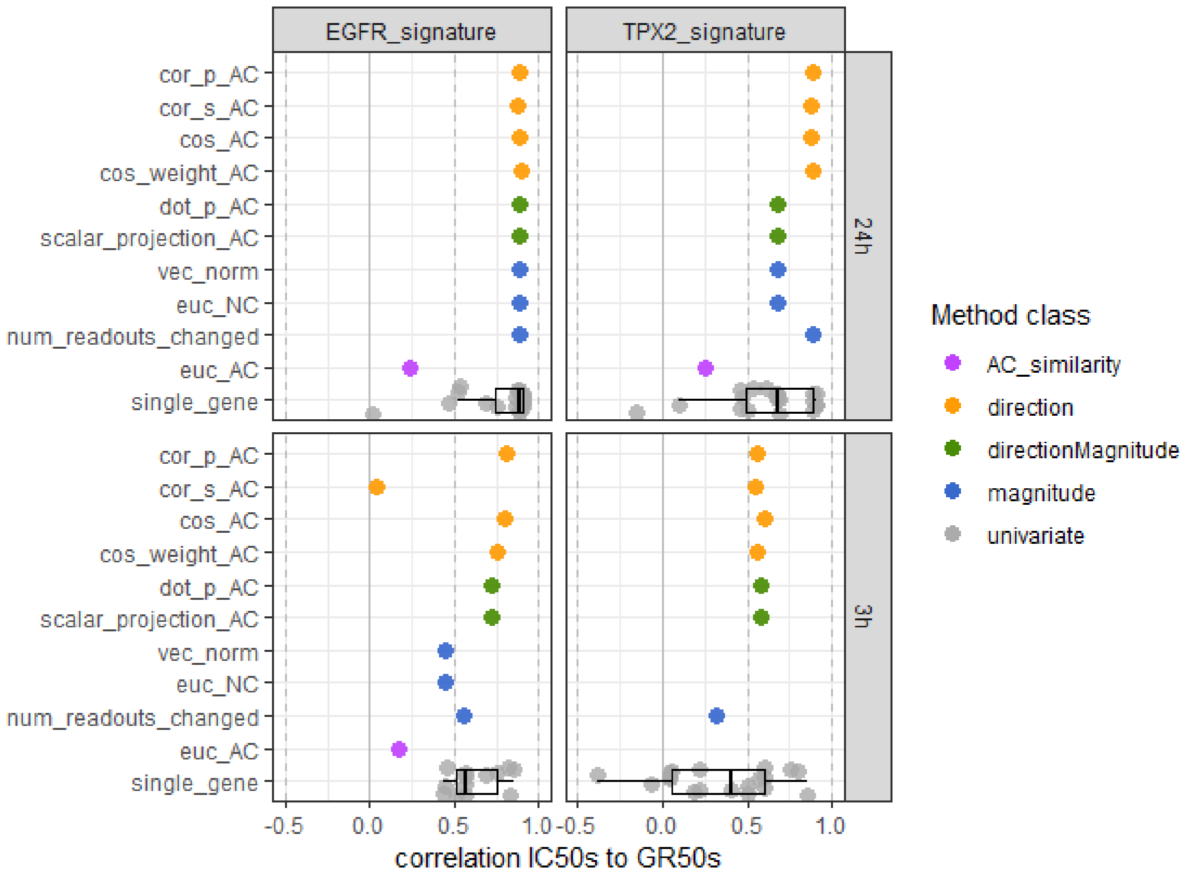

Supplementary Figure 4: Correlation of cell growth inhibition GR_{50}s with all gene and gene-signature EC_{50}s of the EGFR inhibitor dataset.

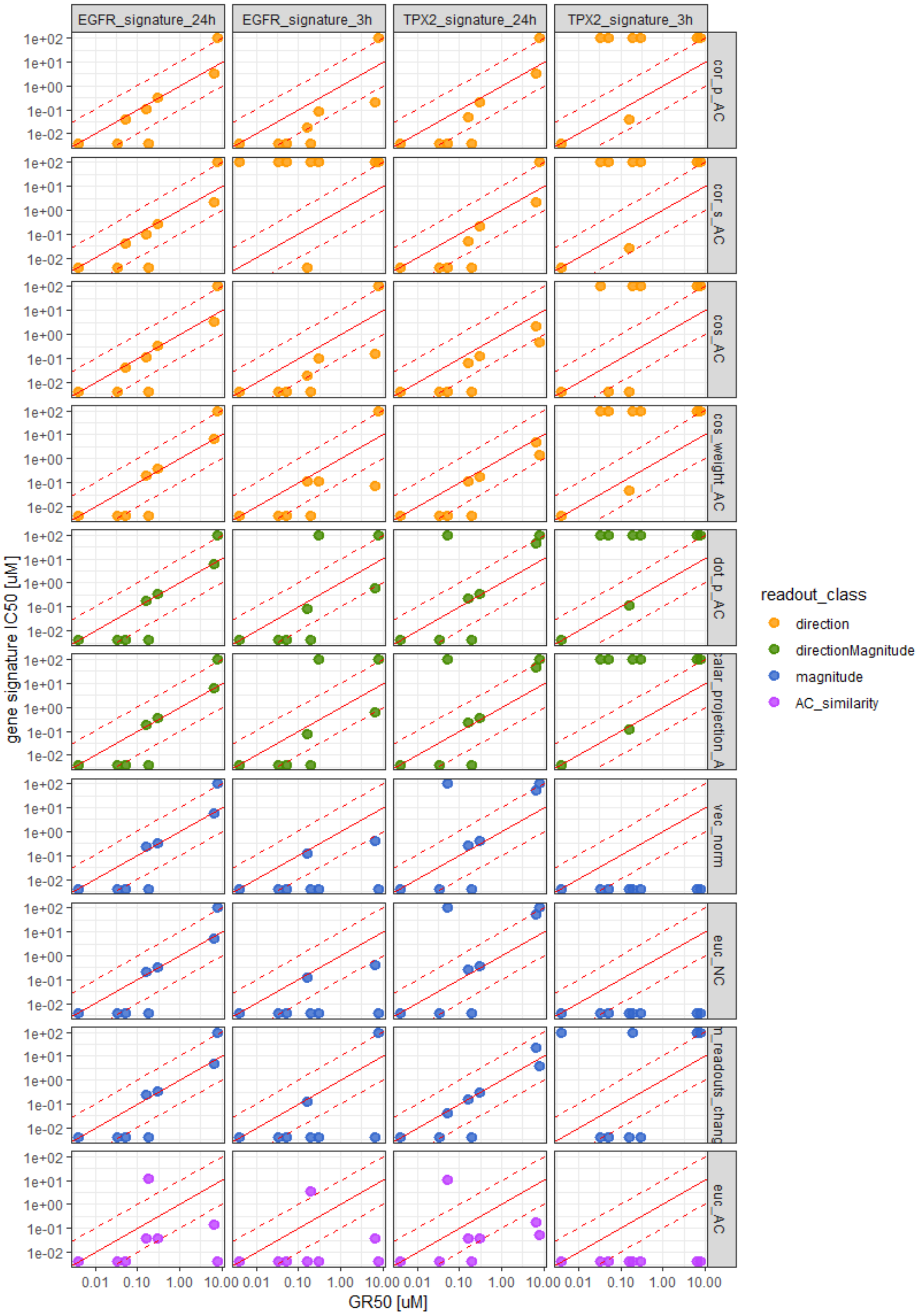

Supplementary Figure 5: Correlation of gene-signature IC_{50}s to GR_{50}s of the EGFR inhibitor dataset.

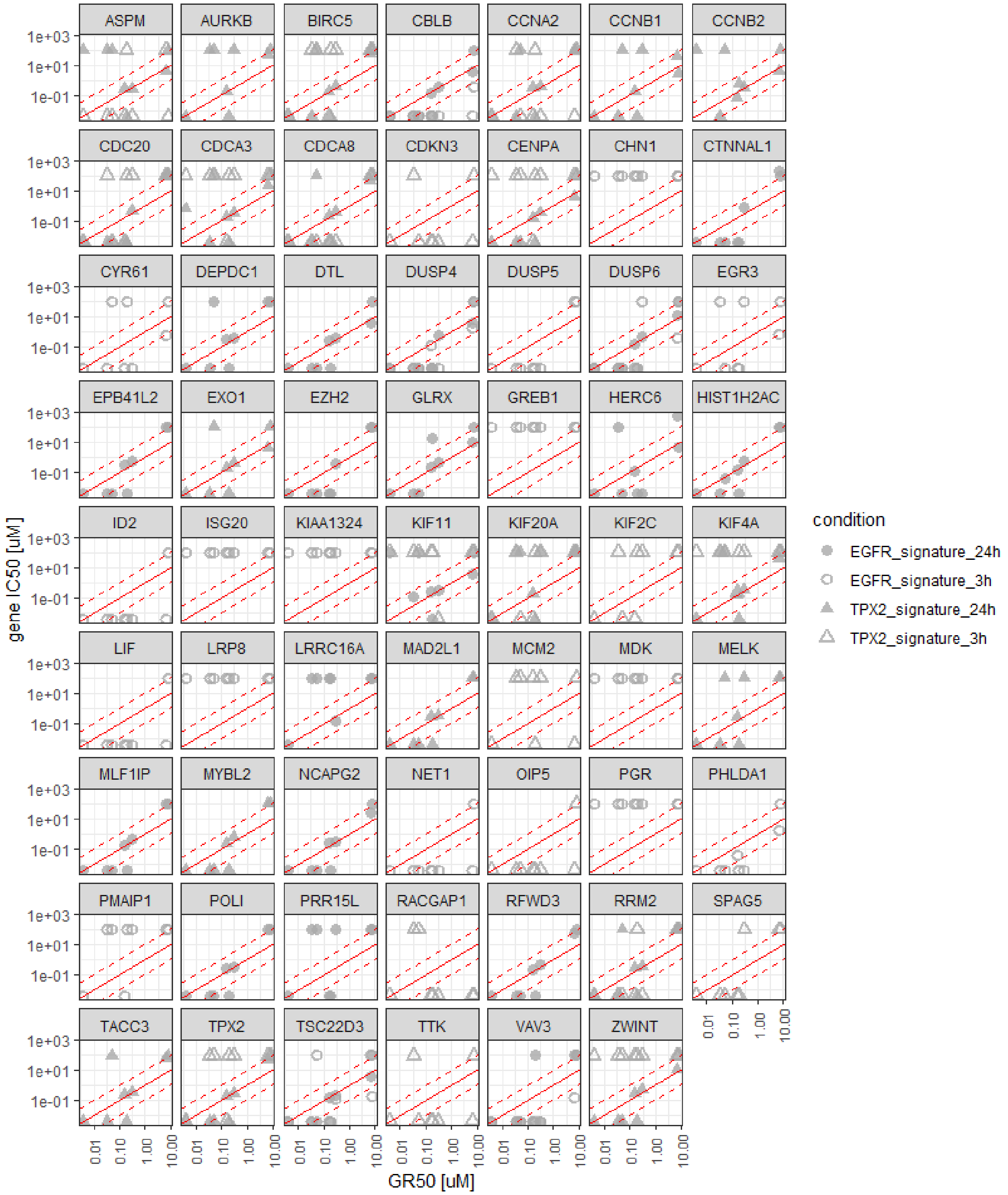

Supplementary Figure 6: Correlation of single gene IC_{50}s to GR_{50}s of the EGFR inhibitor dataset, for the 20 genes most responding to the active control from the EGFR and TPX2 signatures.
